# Low clinical relevance of risky alcohol consumption in a selected group of high adherent HIV-infected patients attended in the United Kingdom

**DOI:** 10.7448/IAS.17.4.19659

**Published:** 2014-11-02

**Authors:** Alicia Gonzalez Baeza, Ana Milinkovic, Alejandro Arenas-Pinto

**Affiliations:** 1 Centre of Sexual Health and HIV Research, University College London Medical School, London, UK; 2 Centre for Sexual Health & HIV Research, Mortimer Market Centre, off Capper Street, London, UK

## Abstract

**Introduction:**

The prevalence of risky alcohol consumption, associated factors and its impact on the brain is not well established in clinically stable HIV patients.

**Materials and Methods:**

Within the PIVOT neurocognitive sub-study, effectively suppressed HIV-infected adults on either standard cART or ritonavir-boosted PI monotherapy completed the Alcohol Use Disorders Identification Test (AUDIT) designed to detect risky alcohol consumption. They also completed a brief neuropsychological assessment (NPZ 5) composed by five measures. For this cross-sectional analysis, we calculated rates of hazardous (AUDIT=8–15) or harmful (AUDIT=16–19) consumption and likely dependence (AUDIT>20). We explored the association between risky alcohol intakes (AUDIT>8) and clinical/demographical variables, conducting logistic regressions when significant association was found (p<.05). Also, the association between cognitive performance and alcohol consumption was calculated and adjusted by potential confounders.

**Results:**

Of the 146 included participants, the majority were male (86.3%), white (81.5%) and educated (mean years on formal education=15, SD=3.9). Average age was 47.6 years (SD=8.7), and 36.3% had risky consumption (29.5% hazardous, 6.2% harmful, 0.7% likely dependence). White ethnicity and male sex were positively associated with risky consumption (Table 1). After adjustments, white ethnicity remained significantly associated with risky consumption (1.64 [95% CI 0.34–2.95]; p=0.013). Better cognitive performance was associated with risky alcohol consumption in the univariate analysis (p<.001). After adjustment by ethnicity, sex and years of education, cognitive performance and risky alcohol consumption maintained significant association (0.45 [95% CI 0.19–0.70] p=0.001).

**Conclusions:**

Despite the substantially high prevalence of risky alcohol consumption, it was not associated with worse adherence, immunological or quality of life measures in this cohort of effectively suppressed patients but prevalence of likely alcohol dependency was extremely low. White patients were more vulnerable to risky consumption. Moreover, positive association between cognitive performance and risky alcohol consumption remained after adjustment. In our study, the association we found between cognitive function and alcohol consumption does not reflect the effect of alcohol dependency on cognition.

**Table 1:** Association between clinical and demographical variables and risky alcohol consumption

	Coef.	Std.Err.	z	p	95% Conf. interval
Ethnicity	1.757	0.639	2.75	0.006	0.503–3.011
Sex	−1.811	0.767	−2.36	0.018	−3.315–0.308
Age	−0.006	0.200	−0.35	0.730	−0.461–0.032
Years of education	−0.001	0.436	−0.03	0.979	−0.086–0.084
Recreational drug use					
In the past	0.820	0.442	1.86	0.063	−0.045–1.687
Currently	0.573	0.468	1.23	0.220	−0.343–1.490
CD4 cell count	0.000	0.001	0.67	0.501	−0.001–0.002
Viral load	−1.063	1.109	−0.96	0.338	−3.238–1.110
Anxiety/depression perception					
Moderately	−0.115	0.383	−0.30	0.762	−0.866–0.634
Extremely	−1.166	1.117	−1.04	0.297	−3.355–1.024
Physical health summary score	−0.175	0.197	−0.98	0.329	−0.527–0.018
Mental health summary score	0.005	0.018	0.28	0.779	−0.034–0.041
Self-reported adherence	−0.165	0.499	−0.33	0.741	−1.143–0.813

